# Static examination of the range of ankle joint dorsiflexion is not related to dynamic foot kinematics

**DOI:** 10.1186/1757-1146-5-S1-P21

**Published:** 2012-04-10

**Authors:** Hannah Jarvis, Christopher J Nester, Peter P Bowden, Richard K Jones, Anmin Liu

**Affiliations:** 1 School of Health, Sport and Rehabilitation Sciences, Centre for Health, Sport and Rehabilitation Sciences Research, University of Salford, Salford, M6 6PU, UK

## Background

Our aim was to (1) investigate whether the range of ankle dorsiflexion measured in a static examination relates to the sagittal plane motion within the foot during walking and (2) investigate the popular clinical theory that individuals with limited ankle joint dorsiflexion (in a static examination [1,2]) will demonstrate increased rearfoot eversion during walking [1-3].

## Materials and method

The static range of ankle joint dorsiflexion was measured with the knee flexed and extended (n=100). Dynamic foot kinematics were measured for the tibia, calcaneus, midfoot, lateral forefoot, medial forefoot and hallux, and 13 parameters derived to characterise foot kinematics (right foot only). The relationship between static range of ankle joint dorsiflexion and sagittal plane motion within the foot during walking was examined using Pearsons correlation. An independent t-test (p<0.05) was used to compare dynamic foot kinematics in subjects exhibiting <10° and >15° of static ankle joint dorsiflexion (n=83, n=7 knee extended, n=40, n=23 knee flexed).

## Results

The range of ankle joint dorsiflexion measured statically was poorly correlated [4] with all 13 parameters describing dynamic foot kinematics (all r values<-0.254, p<0.05). Individuals with <10° of static ankle joint dorsiflexion exhibited less eversion of the calcaneus relative to the tibia between forefoot loading and heel lift (mean, 2.1° eversion motion compared to 4.8° eversion motion (knee extended), and 1.6° eversion motion compared to 3.5° (knee flexed)). Also, there was less plantarflexion of the medial forefoot relative to the midfoot between heel lift and toe off (mean value of 13.1° compared to 18.5°).

## Conclusions

Static assessment of ankle joint dorsiflexion does not appear to relate to dynamic foot kinematics. The differences in foot kinematics in those with <10 or >15 of ankle joint dorsiflexion measured from static examination contradict a key principle of the current clinical paradigm from Root et al [1,2].
